# Supplementary material for: Humidity response in Drosophila olfactory sensory neurons requires the mechanosensitive channel TMEM63
Source: Nat Commun. 2022 Jul 2;13:3814. doi: 10.1038/s41467-022-31253-z (PMC9250499; doi:10.1038/s41467-022-31253-z)
Supplement: Supplementary file 1 — Supplementary Information [file 41467_2022_31253_MOESM1_ESM.pdf]

Supplementary Information

Humidity response in *Drosophila* olfactory sensory neurons requires the mechanosensitive channel TMEM63

Songling Li[1,2], Bingxue Li[1,2], Li Gao[1], Jingwen Wang[1], Zhiqiang Yan[1,2]*

[1] State Key Laboratory of Medical Neurobiology and MOE Frontiers Center for Brain Science, Ministry of Education Key Laboratory of Contemporary Anthropology, School of Life Sciences, Fudan University, Shanghai 200438, China.

[2] Institute of Molecular Physiology, Shenzhen Bay Laboratory, Shenzhen 518132, China

*Correspondence to: Zhiqiang Yan (zqyan@szbl.ac.cn)

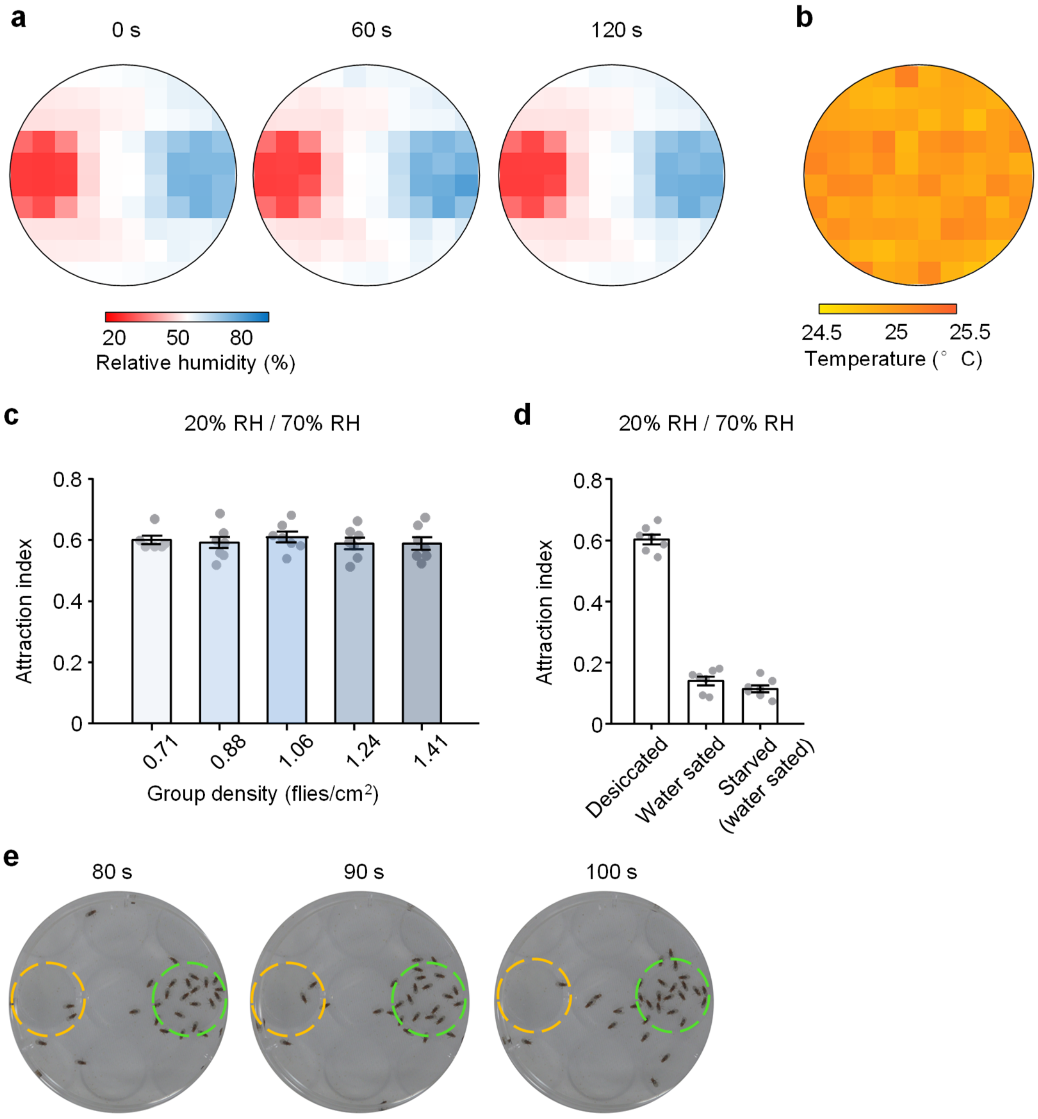

Supplementary Fig. 1

Moisture attraction depends on the humidity gradient and internal state but not group densities. a, Humidity distribution in the Petri dish arena over the 120 s experimental period. Color intensity represents the relative humidity ranging from 20% to 80% RH. **b**, Temperature distribution in the Petri dish arena. **c**, Attraction index of w^{1118} flies to 70% RH tested for five group densities. Group densities 0.71, 0.88, 1.06, 1.24, 1.41 correspond to 20, 25, 30, 35, 40 flies introduced to an assay. n = 6, 8, 7, 7, 7 assays. **d**, Attraction indexes of w^{1118} flies that are desiccated for 6 h, starved overnight or sated. n = 7 for each group. For **c, d**, data are mean ± SEM. **e**, Representative images showing the aggregation of flies above the 70% RH area at time points 80 s, 90 s and

100 s. The yellow dashed circle denotes the area above 20% RH, and the green circle indicates the 70% RH region.

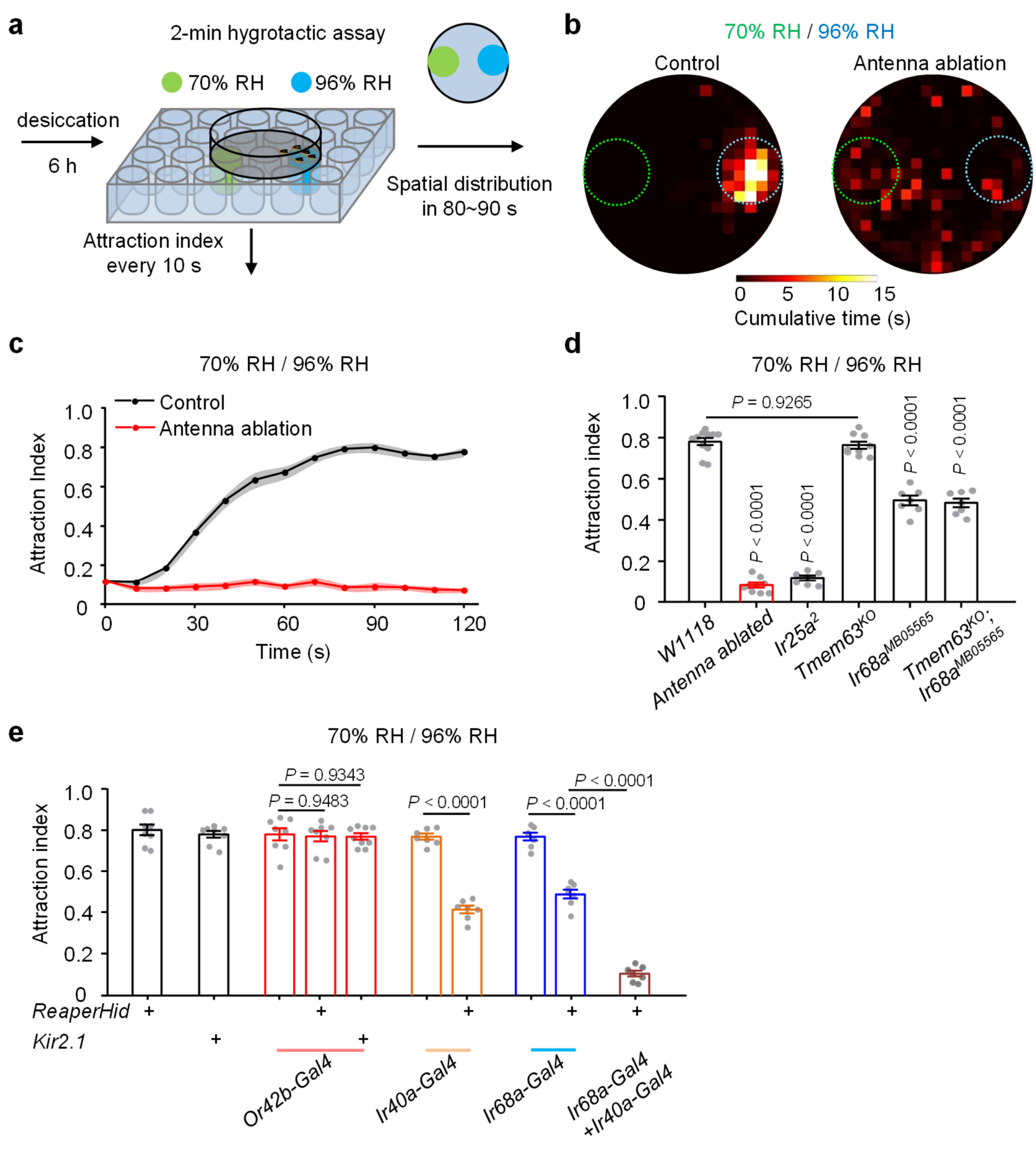

Supplementary Fig. 2

Or42b neurons and *Tmem63* are dispensable for water seeking behavior in the 70% to 96% RH gradient. a, Schematic diagram depicting the water-induced attraction assay. The green region represents 70% RH generated by saturated NaCl solution, and the blue region denotes 96% RH produced by distilled water. A total of 20-40 flies were used per assay. **b**, Spatial distribution of control flies (left) and flies with the antennae removed (right) assayed in 70% to 96% RH gradient. w^{1118} flies were used as control flies. Control, n = 24 flies; Antenna ablation, n = 23. The green dashed circle denotes the area above 70% RH, and the blue circle indicates the 96% RH region. Color intensity represents the cumulative time the flies spent on each pixel during 80-90 s

after the onset of the assay. **c**, The time course indicating the attraction indexes of control and antenna-ablated flies. Control, n = 11 assays; Antenna ablation, n = 9. Data points are mean values and shaded area represents ± SEM. **d**, The effects of different mutations on the water-induced attraction. n = 11, 9, 7, 9, 7, 7 assays. **e**, Attraction index for 96% RH after ablating or inactivating different neuron classes. n = 8, 8, 8, 8, 9, 7, 7, 7, 7, 7 assays. Data are mean ± SEM. Two-tailed unpaired t test for two groups, one-way ANOVA followed by Dunnett's test for multiple comparison.

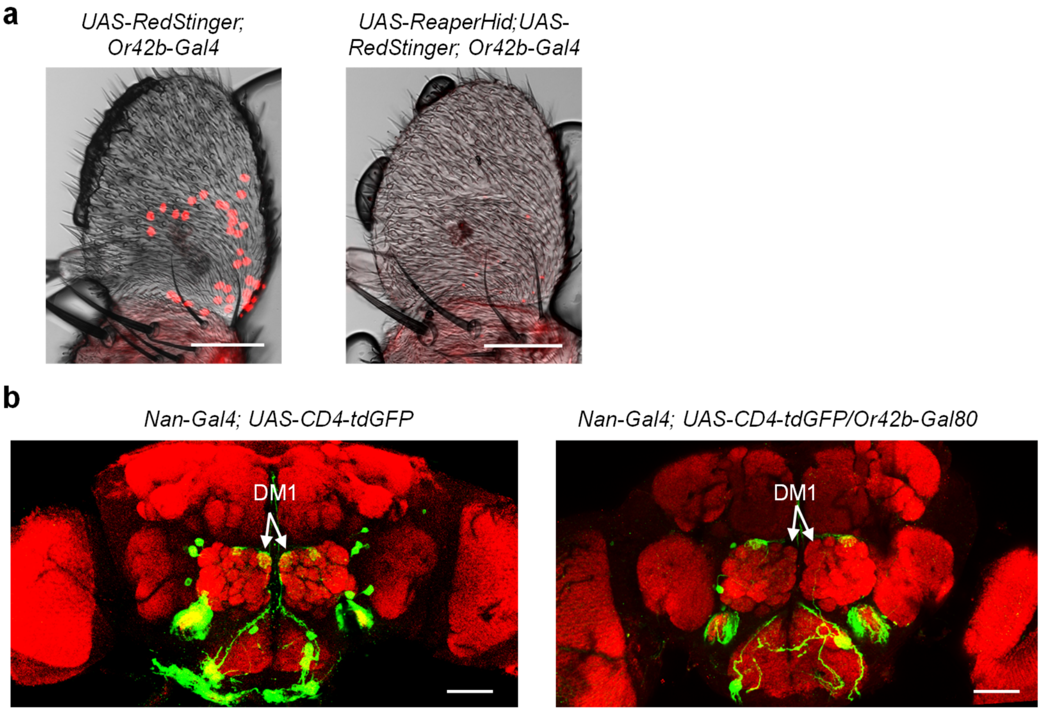

Supplementary Fig. 3

Expression of *Nan-Gal4* in the antennal lobe and confirmation of the efficiency of ReaperHid. **a**, Expression of Reaper-Hid eliminated the *Or42b-Gal4*-expressing neurons in the antenna. Scale bar, 50 µm. **b**, *Or42b-Gal80* inhibits the staining of *Nan-Gal4* in DM1 glomerulus, indicating an overlap between the two drivers. Scale bar, 50 µm. Each result was repeated three times.

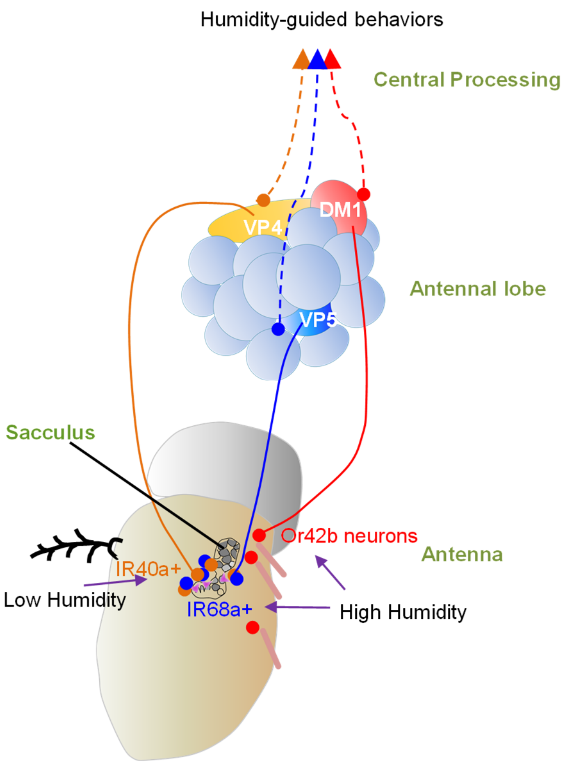

Supplementary Fig. 4

Hypothetical wiring model of hygrosensory system. Certain groups of humidity sensitive neurons work in parallel in humidity sensation.

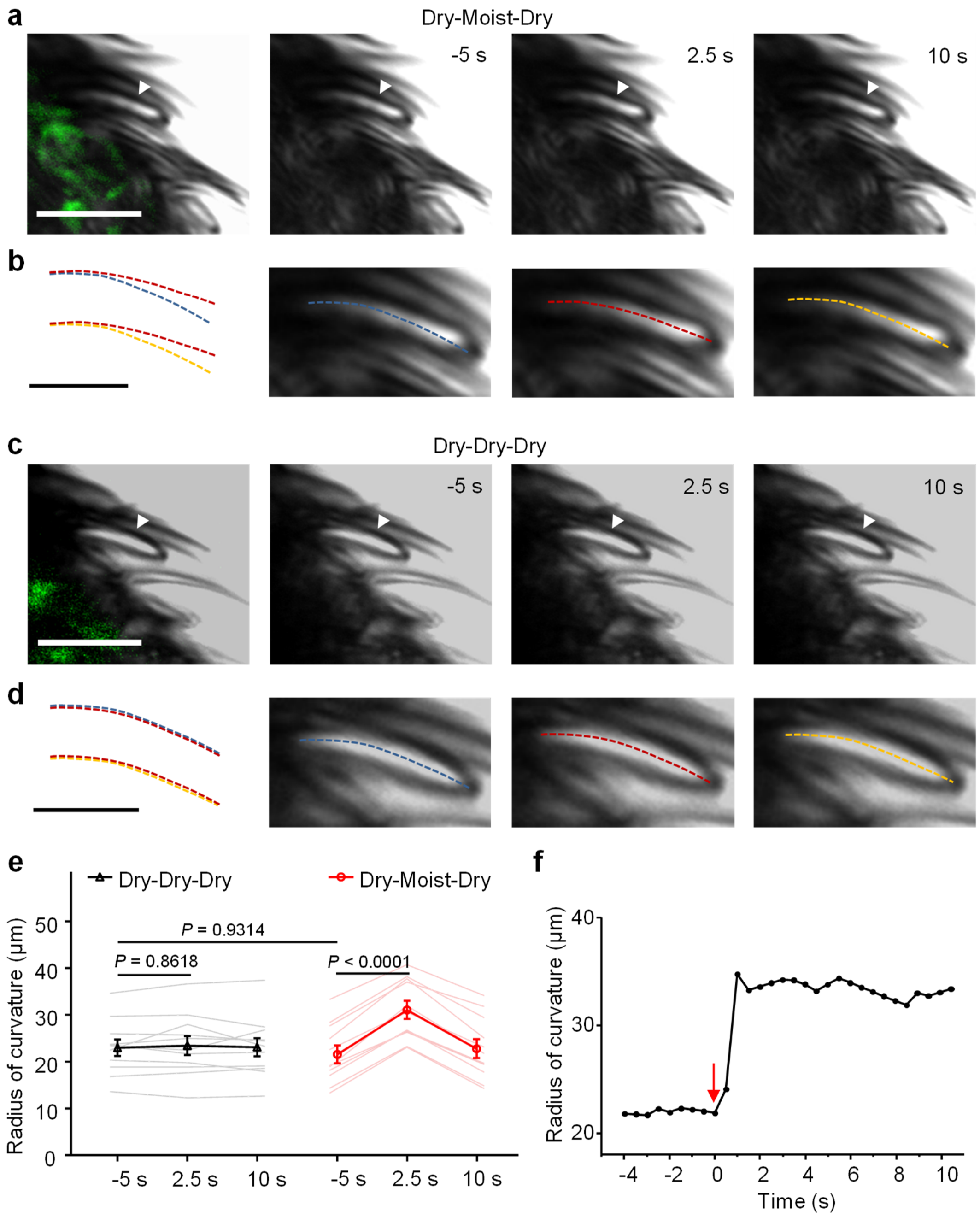

Supplementary Fig. 5

The deformation of large basiconic sensilla in response to humidity change. a, Time-lapse images showing humidity-induced curvature changes in basiconic sensilla that is not labeled by *Or42b-Gal4*, the monitored sensillum is indicated by a white arrowhead. Scale bar, 10 μm. **b**, Zoomed in images of the sensillum indicated in **a**, dashed lines represent the curves fitted to the shape of the sensillum. Scale bar, 5 μm. **c**, Images showing morphology of the basiconic sensillum upon exposure to dry

airflows. Scale bar, 10 µm. **d**, Zoomed in images of the sensillum indicated in **c**. Scale bar, 5 µm. **a**-**d**, Representative images from over three independent replicated experiments. **e**, Summary of curvature change of GFP negative basiconic sensilla under dry-dry-dry or dry-moist-dry air stimuli. n = 11 sensilla for both groups. Data are mean ± SEM. Two-way ANOVA followed by Sidak's post hoc test. **f**, Dynamic changes of ab1 sensilla curvature in response to a moist airflow lasting for 10 s. The red arrow indicates the time point of switching from dry air to moist air.

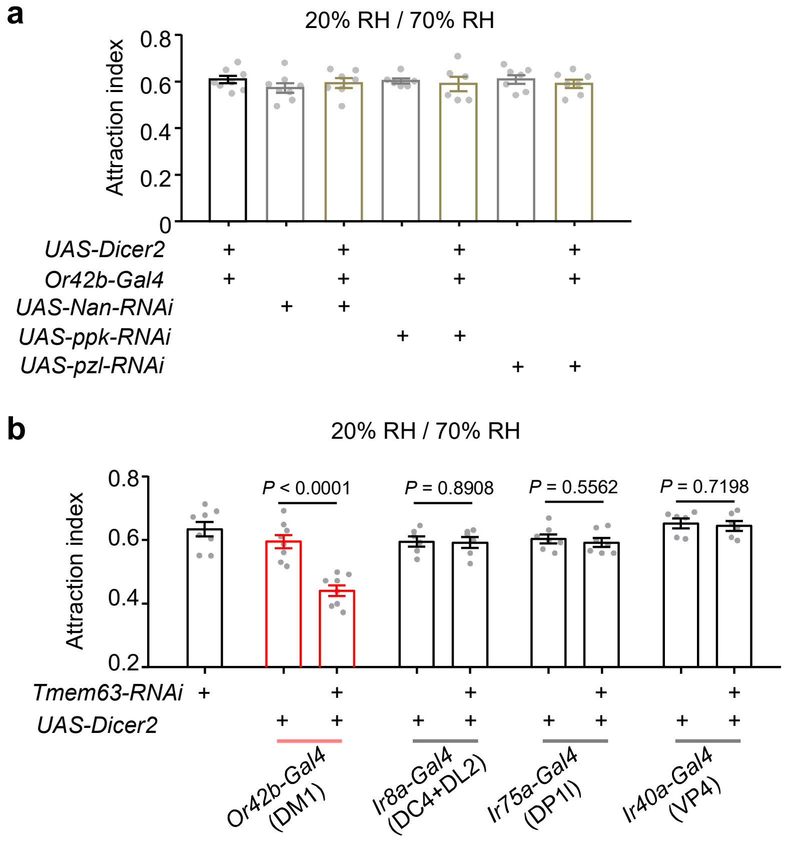

Supplementary Fig. 6

Behavioral screening by RNAi-mediated gene silencing. a, Behavioral screening for genes involved in moisture attraction behavior. *UAS-RNAi* lines were crossed to *Or42b-Gal4*. n = 8, 8, 7, 6, 6, 7, 7 assays. **b**, Screening for humidity responsive neurons that are responsible for *Tmem63*-dependent behavioral phenotype. *IR8a-Gal4* is a marker for both IR75abc-expressing OSNs and IR64a positive OSNs[1]. n = 8, 8, 8, 6, 6, 7, 7, 6, 6 assays. Two-tailed unpaired t test. Data are mean ± SEM.

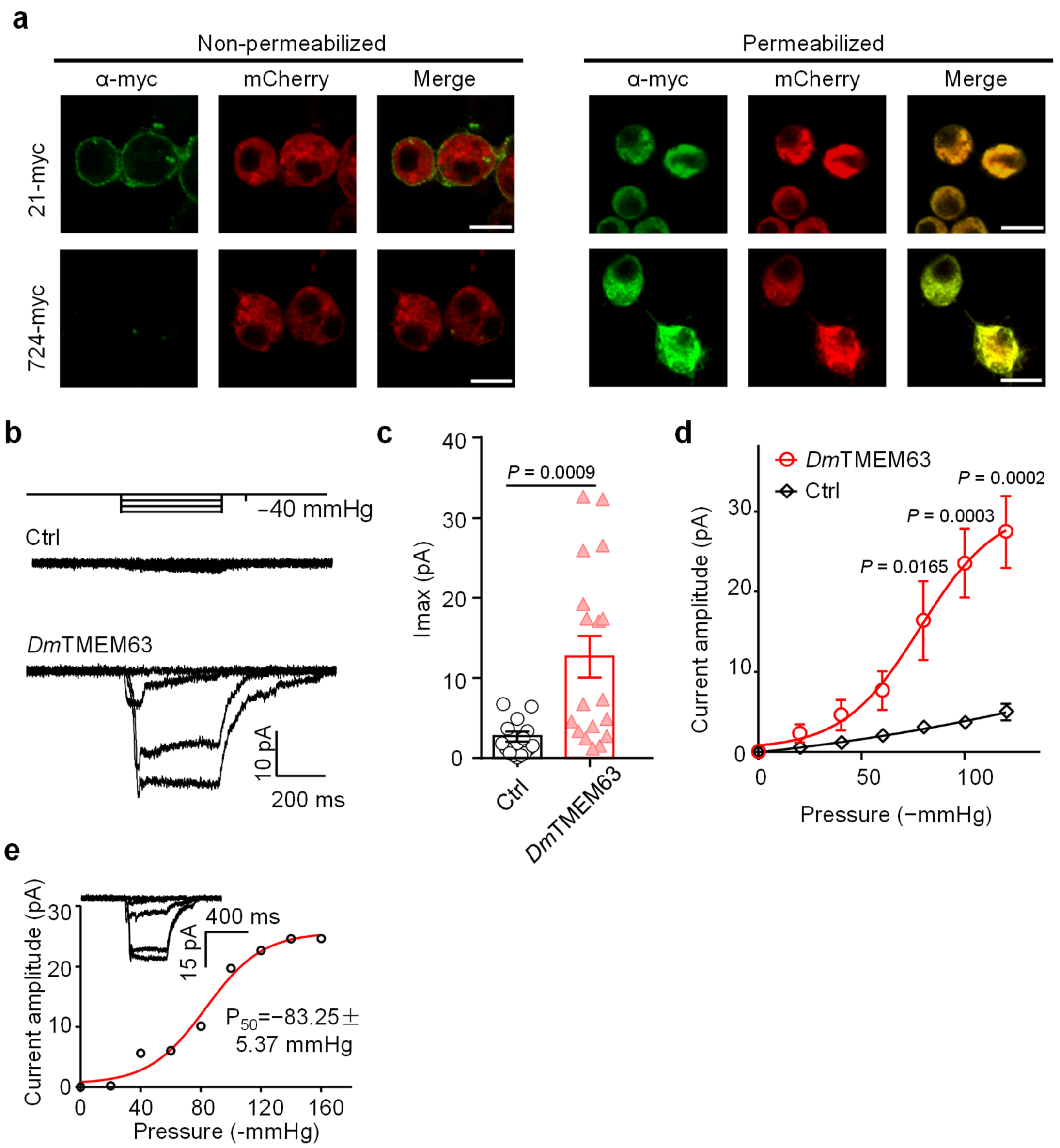

Supplementary Fig. 7

***Dm*TMEM63 confers stretch-activated currents to S2 cells.** **a**, Representative images of myc staining in Tmem63-mCherry-transfected S2 cells. Myc-tags were inserted in the N-terminal region (upper panels) or C-terminal region (lower panels) of *Dm*TMEM63. Each result was repeated three times. Scale bar, 10 μm. **b**, Representative outside-out current traces elicited by negative pressure at -60 mV in S2 cells expressing empty vector (upper) and *Dm*TMEM63-GFP (lower). **c**, Maximal current responses to membrane stretch of cells transfected with indicated genes. n = 16, 18 cells. Two-tailed unpaired t test. **d**, Average peak current-pressure relationship of stretch-activated

currents in S2 cells expressing *Dm*TMEM63 or empty vector. n = 9 cells for each group. Two-tailed unpaired t test. Data are mean ± SEM fitted with a Boltzmann equation. **e**, To estimate the P_{50} of *Dm*TMEM63, stretch-activated currents from one recording (inset) were fitted with the Boltzmann equation. Inset: current traces at −0, −40, −80, −120 and −160 mmHg recorded from one *Dm*TMEM63-transfected cell at holding potential of −60 mV.

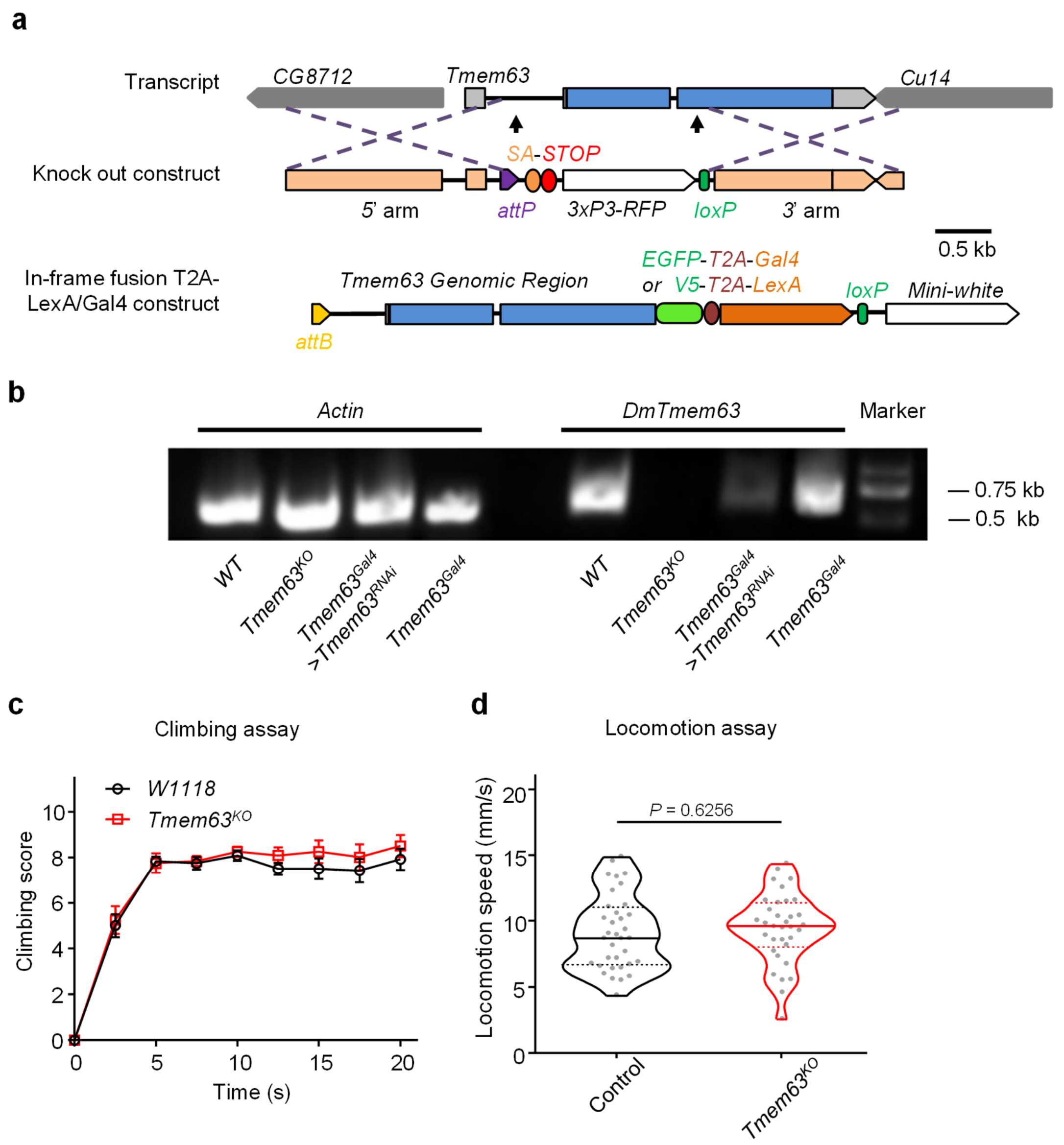

Supplementary Fig. 8

Generation of *Tmem63* reporter and mutant alleles. a, Targeting strategy for the generation of the *Tmem63^{KO}* mutant allele and the generation of knock-in *Tmem63^{LexA}* and *Tmem63$^{EGFP\text{-}Gal4}$* alleles. The black arrows under the transcript indicate two sgRNA target sites. We first obtained the *Tmem63^{KO}* mutant allele with an attp site introduced to the first intron, then reporter constructs were integrated into the attP site to generate the corresponding reporter lines, further details can be found in methods. **b**, RT-PCR confirms the *Tmem63^{KO}* mutation as a null allele and the knock down efficiency of *Tmem63^{RNAi}*. Each result was reproducible in three independent experiments. **c**, Climbing score of *w^{1118}* and *Tmem63^{KO}* mutant flies in a 20 s climbing assay[2]. n = 12

assays for both groups. Data are mean ± SEM. **d**, Average locomotion speed of control and *Tmem63*^{KO} mutant flies during a 2 min period. n = 35 and 34 flies. Two-tailed unpaired t test. For each violin plot, the middle line denotes the median, and the top and bottom lines indicate the 75th and 25th percentile.

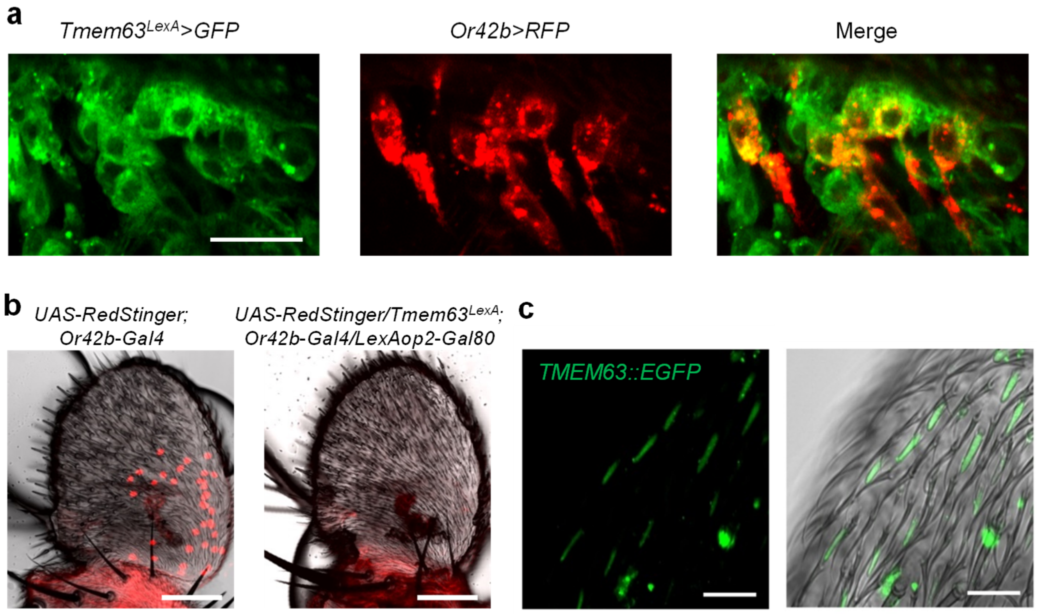

Supplementary Fig. 9

Overlap between *Tmem63^LexA^* and *Or42b-Gal4* drivers. a, Double labeling of *Tmem63^LexA^* and *Or42b-Gal4* in OSNs. Scale bar, 10 µm. Genotype: *UAS-mCD8-RFP,LexAop2-mCD8-GFP*; *Tmem63^LexA^*/+; *Or42b-Gal4*/+. **b**, Nuclear expression of *Or42b-Gal4* (left) in antennae. *Tmem63>Gal80* inhibits the expression of *Or42b-Gal4* in antennal neurons (right). Scale bar, 50 µm. **c**, Fluorescent immunostaining of GFP labels the basiconic sensilla in the antenna of *Tmem63^EGFP^* flies. Scale bar, 10 µm. Each result was repeated three times.

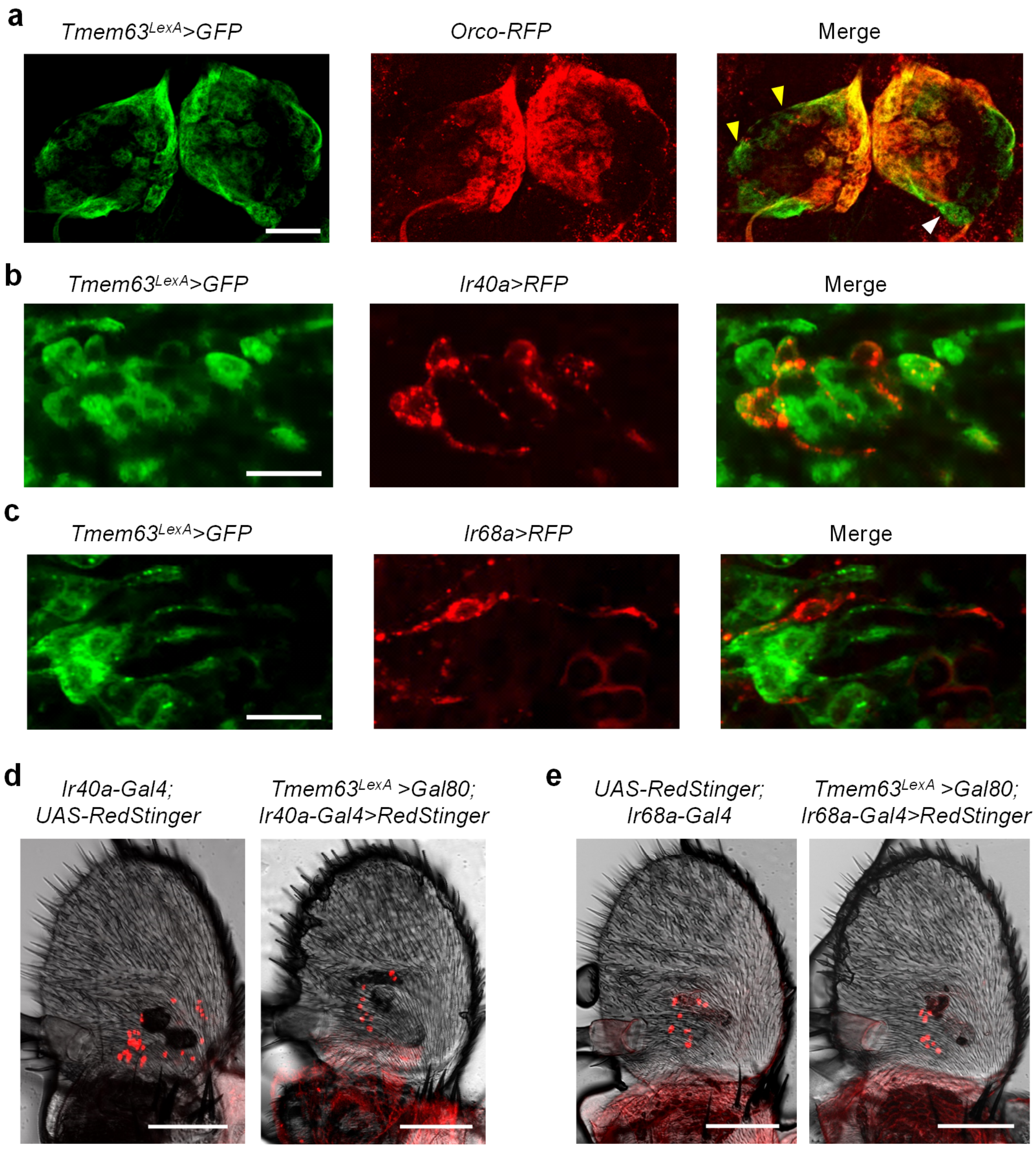

Supplementary Fig. 10

Co-expression of *Tmem63*^{LexA} with *Or*- and *Ir*-drivers. a, Double labeling with *Orco-RFP* in the antennal lobe. The yellow arrowheads indicate *Tmem63*^{LexA} labeling in two glomeruli innervated by Ir-expressing neurons. The white arrowhead denotes the V glomerulus. Scale bar, 50 µm. Genotype: *LexAop2-mCD8-GFP*; *Tmem63*^{LexA}/+; *Orco-RFP*/+. **b**, **c** Double labeling with markers for sacculus dry cells and moist cells. Scale bar, 10 µm. Genotype: U*AS-mCD8-RFP,LexAop2-mCD8-GFP*; *Tmem63*^{LexA}/*Ir40a-Gal4* in **b**, *UAS-mCD8-RFP,LexAop2-mCD8-GFP*; *Tmem63*^{LexA}/+; *Ir68a-Gal4*/+ in **c**. **d**, Nuclear expression of *Ir40a-Gal4* (left) in antennae. *Tmem63>Gal80* inhibits the

fluorescence in most *Ir40a*-expressing neurons (right). Scale bar, 50 µm. Genotype: *Tmem63^{LexA}*/*Ir40a-Gal4*; *LexAop-Gal80* /*UAS-RedStinger*. **e**, Nuclear expression of *Ir68a-Gal4* (left) in antennae. *Tmem63>Gal80* has no effect on the labeling by *Ir68a-Gal4* (right). Scale bar, 50 µm. Genotype: *Tmem63^{LexA}*/*UAS-RedStinger*; *LexAop-Gal80*/*Ir68a-Gal4*. **a**-**e**, Representative images of three biological replicates.

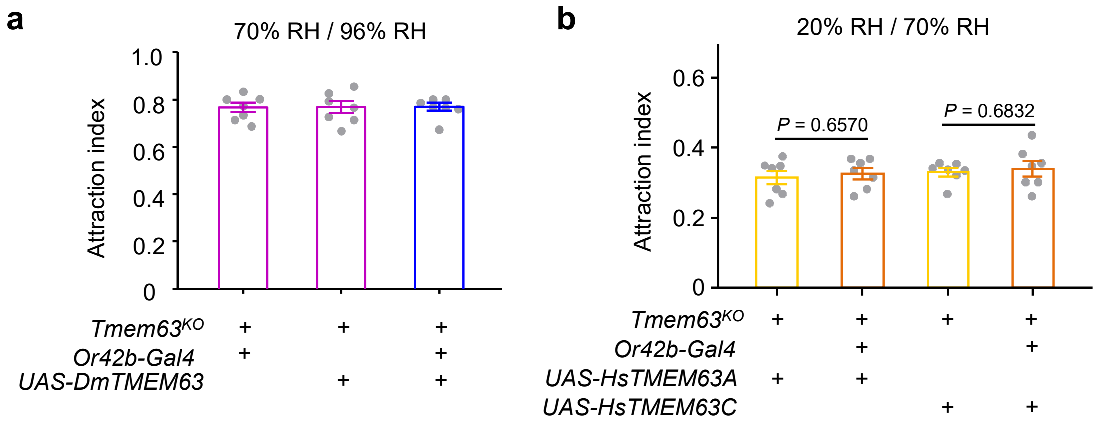

Supplementary Fig. 11

The effect of expressing TMEM63 homologs on the phenotype of *Tmem63^{KO}* mutants. a, Water-induced attraction to 96% RH in *Dm*TMEM63 rescue and rescue control flies. n = 7 assays for each group. **b**, Attraction index of human rescue (TMEM63A and TMEM63C) and rescue control groups tested in the 20% to 70% RH gradient. n = 7 assays for each group. Two-tailed unpaired t test. Data are mean ± SEM.

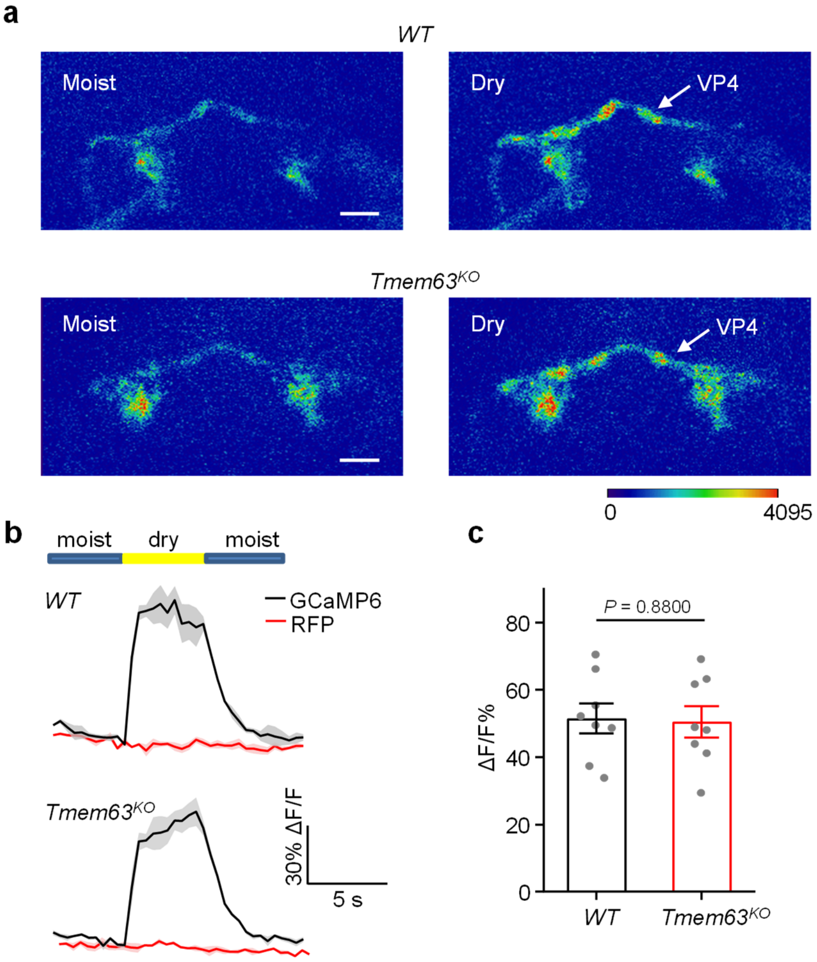

Supplementary Fig. 12

Dry response of IR40a neurons is independent of *Tmem63*. a, Pseudocolored images showing GCaMP6m fluorescence of neuronal projections in VP4 glomerulus from *wild type* (upper) and *Tmem63^{KO}* (lower) flies in response to dry air. Scale bar, 20 µm. Genotypes are *wild type*: *Ir40a-Gal4*/+; *UAS-GCaMP6m,UAS-tdTomato*/+. *Tmem63^{KO}*: *Ir40a-Gal4,Tmem63^{KO}*/*Tmem63^{KO}*; *UAS-GCaMP6m,UAS-tdTomato*/+. Representative images from over three independent replicated experiments. **b**, Representative traces for **a**. Average $\Delta F/F_0$ for three dry air stimulation cycles in the same antennal lobe was shown. **c**, Statistical analysis of dry response in IR40a neurons of *wild type* and *Tmem63^{KO}* flies. n = 8 flies for each group. Two-tailed unpaired t test. Data are mean ± SEM.

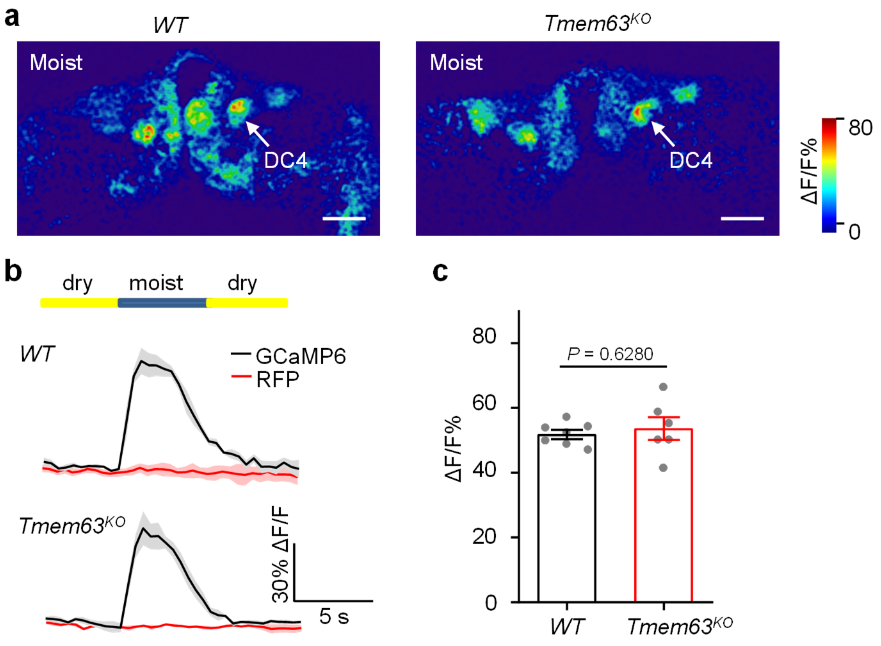

Supplementary Fig. 13

Moist response of IR64a neurons is independent of *Tmem63*. **a**, Pseudocolored images showing GCaMP6m responses ($\Delta F/F_0$) of neuronal projections in DC4 glomerulus from *wild type* and *Tmem63^{KO}* flies in response to moist air. Scale bar, 20 µm. Genotypes are *wild type*: *Pebbled-Gal4*/y; *UAS-GCaMP6m,UAS-tdTomato*/+. *Tmem63^{KO}*: *Pebbled-Gal4*/y;*Tmem63^{KO}*/*Tmem63^{KO}*; *UAS-GCaMP6m,UAS-tdTomato*/+. Representative images from over three independent replicated experiments. **b**, Representative traces for **a**. Average $\Delta F/F_0$ for three dry air stimulation cycles in the same antennal lobe was shown. **c**, Statistical analysis of dry response in IR40a neurons of *wild type* and *Tmem63^{KO}* flies. n = 7, 6 flies. Two-tailed unpaired t test. Data are mean ± SEM.

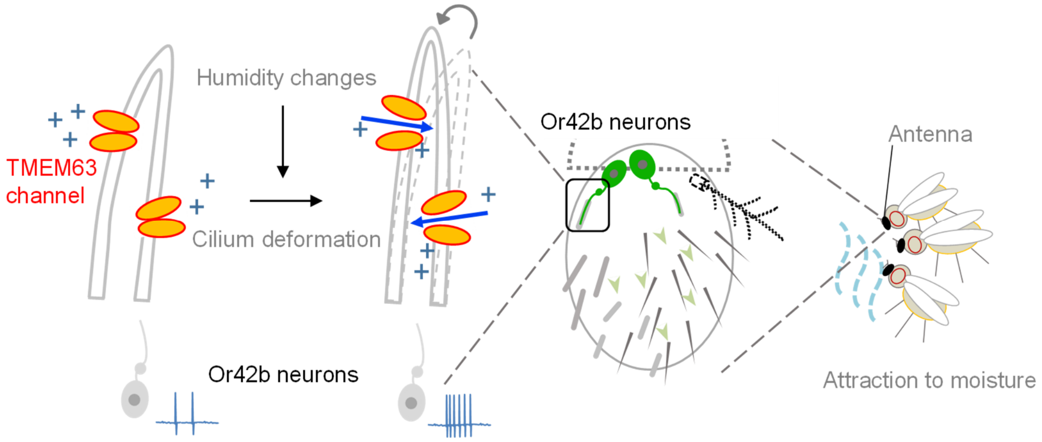

Supplementary Fig. 14

Proposed hygrosensory transduction model for Or42b neurons. Humidity changes are first converted to membrane deformation in the sensory cilia of the Or42b neurons; this imbalance of membrane force opens the mechanosensitive channel TMEM63, thus leading to the physiological and behavioral response to changing humidity levels.

Supplementary References

1. Silbering, A. F. et al. Complementary Function and Integrated Wiring of the Evolutionarily Distinct Drosophila Olfactory Subsystems. *J. Neurosci.* **31**, 13357-13375 (2011).

2. Sun, Y. et al. TRPA channels distinguish gravity sensing from hearing in Johnston's organ. *Proc. Natl. Acad. Sci. USA* **106**, 13606-13611 (2009).
