# Supplementary material for: Humidity response in Drosophila olfactory sensory neurons requires the mechanosensitive channel TMEM63
Source: Nat Commun. 2022 Jul 2;13:3814. doi: 10.1038/s41467-022-31253-z (PMC9250499; doi:10.1038/s41467-022-31253-z)
Supplement: Supplementary file 3 — Description of Additional Supplementary Files [file 41467_2022_31253_MOESM3_ESM.pdf]

Description of Additional Supplementary Files

Supplementary Movie 1. Moisture attraction behavior of w^{1118} flies in 20% (yellow circle) to 70% (green circle) RH gradient.

Supplementary Movie 2. Antenna ablated w^{1118} flies showed no moisture attraction behavior in 20% (yellow circle) to 70% (green circle) RH gradient.
