# Supplementary material for: Humidity response in Drosophila olfactory sensory neurons requires the mechanosensitive channel TMEM63
Source: Nat Commun. 2022 Jul 2;13:3814. doi: 10.1038/s41467-022-31253-z (PMC9250499; doi:10.1038/s41467-022-31253-z)
Supplement: Supplementary file 6 — Reporting Summary [file 41467_2022_31253_MOESM6_ESM.pdf]

nature portfolio

Corresponding author(s): Zhiqiang Yan

Last updated by author(s): Apr 15, 2022

# Reporting Summary

Nature Portfolio wishes to improve the reproducibility of the work that we publish. This form provides structure for consistency and transparency in reporting. For further information on Nature Portfolio policies, see our Editorial Policies and the Editorial Policy Checklist.

## Statistics

For all statistical analyses, confirm that the following items are present in the figure legend, table legend, main text, or Methods section.

| n/a | Confirmed | |
|---|---|---|
| ☐ | ☒ | The exact sample size (*n*) for each experimental group/condition, given as a discrete number and unit of measurement |
| ☐ | ☒ | A statement on whether measurements were taken from distinct samples or whether the same sample was measured repeatedly |
| ☐ | ☒ | The statistical test(s) used AND whether they are one- or two-sided<br>*Only common tests should be described solely by name; describe more complex techniques in the Methods section.* |
| ☐ | ☒ | A description of all covariates tested |
| ☐ | ☒ | A description of any assumptions or corrections, such as tests of normality and adjustment for multiple comparisons |
| ☐ | ☒ | A full description of the statistical parameters including central tendency (e.g. means) or other basic estimates (e.g. regression coefficient) AND variation (e.g. standard deviation) or associated estimates of uncertainty (e.g. confidence intervals) |
| ☐ | ☒ | For null hypothesis testing, the test statistic (e.g. *F*, *t*, *r*) with confidence intervals, effect sizes, degrees of freedom and *P* value noted<br>*Give P values as exact values whenever suitable.* |
| ☒ | ☐ | For Bayesian analysis, information on the choice of priors and Markov chain Monte Carlo settings |
| ☒ | ☐ | For hierarchical and complex designs, identification of the appropriate level for tests and full reporting of outcomes |
| ☒ | ☐ | Estimates of effect sizes (e.g. Cohen's *d*, Pearson's *r*), indicating how they were calculated |

*Our web collection on statistics for biologists contains articles on many of the points above.*

## Software and code

Policy information about availability of computer code

| | |
|---|---|
| Data collection | Confocal microscopy and calcium imaging were performed with FV10-ASW 4.2 software from FV1200 (Olympus).Electrophysiological signals were obtained and stored using the Clampex 10.5 software (Molecular Devices). |
| Data analysis | Animal behavior analysis was performed with Flytracker (http://www.vision.caltech.edu/Tools/FlyTracker) in Matlab (R2018b). Spatial distribution of flies was analyzed using Matlab (R2018b) based on generic codes from Plotly (https://plotly.com/matlab/2D-Histogram). Electrophysiological data were analyzed with pCLAMP 10.5 programs (Molecular Devices). Fiji/ImageJ v1.53c and Kappa plugin (https://github.com/brouhardlab/Kappa) were used to analyze imaging data. Statistical analysis were performed in GraphPad Prism 7. |

For manuscripts utilizing custom algorithms or software that are central to the research but not yet described in published literature, software must be made available to editors and reviewers. We strongly encourage code deposition in a community repository (e.g. GitHub). See the Nature Portfolio guidelines for submitting code & software for further information.

## Data

Policy information about availability of data

All manuscripts must include a data availability statement. This statement should provide the following information, where applicable:

- Accession codes, unique identifiers, or web links for publicly available datasets
- A description of any restrictions on data availability
- For clinical datasets or third party data, please ensure that the statement adheres to our policy

All data generated in this study are available within the article and its Supplementary Information files. Source data are provided with this paper.

# Field-specific reporting

Please select the one below that is the best fit for your research. If you are not sure, read the appropriate sections before making your selection.

☒ Life sciences ☐ Behavioural & social sciences ☐ Ecological, evolutionary & environmental sciences

For a reference copy of the document with all sections, see nature.com/documents/nr-reporting-summary-flat.pdf

# Life sciences study design

All studies must disclose on these points even when the disclosure is negative.

| | |
|---|---|
| Sample size | No statistical methods to pre-determine sample size were used. Sample sizes were based on the current standard in the field and previous studies (Knecht et al., 2016; Frank et al., 2017). |
| Data exclusions | No data points were excluded. |
| Replication | The key findings of the paper were confirmed by multiple complementary experiments. All attempts at replication were successful. At least three independent biological replicates and experimental findings were reliably reproduced. |
| Randomization | Experimental groups were allocated based on genotypes. For in vivo experiments, flies were randomly selected from each experimental group. For sensilla imaging, multiple regions of interest were randomly selected for image acquisition. For electrophysiological recording, S2 cells expressing the GFP reporter were randomly picked. |
| Blinding | Experimenters were not blinded to the genotype of flies and group allocation during data collection and analysis. Experimental manipulation due to implicit bias was not possible without actively tampering with the raw data which are mostly videos and confocal images. |

# Reporting for specific materials, systems and methods

We require information from authors about some types of materials, experimental systems and methods used in many studies. Here, indicate whether each material, system or method listed is relevant to your study. If you are not sure if a list item applies to your research, read the appropriate section before selecting a response.

## Materials & experimental systems

| n/a | Involved in the study | |
|---|---|---|
| ☐ | ☒ | Antibodies |
| ☐ | ☒ | Eukaryotic cell lines |
| ☒ | ☐ | Palaeontology and archaeology |
| ☐ | ☒ | Animals and other organisms |
| ☒ | ☐ | Human research participants |
| ☒ | ☐ | Clinical data |
| ☒ | ☐ | Dual use research of concern |

## Methods

| n/a | Involved in the study | |
|---|---|---|
| ☒ | ☐ | ChIP-seq |
| ☒ | ☐ | Flow cytometry |
| ☒ | ☐ | MRI-based neuroimaging |

## Antibodies

| | |
|---|---|
| Antibodies used | Mouse anti-myc-tag (Cell Signaling, 2276) 1:200; Rabbit anti-GFP (Proteintech, 50430-2-AP) 1:200; Mouse anti-nc82 (Developmental Studies Hybridoma Bank, nc82) 1:20; Donkey anti-rabbit Alexa488 (Jackson ImmunoResearch, 711-545-152) 1:500; Goat anti-mouse Alexa488 (Jackson ImmunoResearch, 115-545-003) 1:500; Donkey anti-mouse Alexa647 (Jackson ImmunoResearch, 715-605-151) 1:500. |
| Validation | Mouse anti-nc82 antibody has been successfully used in immunocytochemical analysis of the fly brain in previous studies (Benton et al., 2009; Silbering et al., 2011; Knecht et al., 2017). All primary antibodies are commercially obtained and validated by the manufacturer, with detailed validation analysis and relevant citations on the manufacturer's website:<br>Mouse anti-myc-tag (Cell Signaling, https://www.cellsignal.com/products/primary-antibodies/myc-tag-9b11-mouse-mab/2276),<br>Rabbit anti-GFP (Proteintech, https://www.ptgcn.com/Products/eGFP-Antibody-50430-2-AP.htm),<br>Mouse anti-nc82 (DSHB, https://dshb.biology.uiowa.edu/nc82). |

## Eukaryotic cell lines

Policy information about cell lines

| | |
|---|---|
| Cell line source(s) | Schneider 2 cells (S2 cells) were obtained from Lei Zhang lab (Shanghai Institute of Biochemistry and Cell Biology, Shanghai, China) (ATCC Cat# CRL-1963, RRID:CVCL_Z232). |

| | |
|---|---|
| Authentication | Visual inspection was used to confirm the morphology of each cell line (compared to pictures in one previous study; https://doi.org/10.1016/j.cell.2015.08.024). |
| Mycoplasma contamination | All cell lines tested negative for mycoplasma contamination. |
| Commonly misidentified lines (See ICLAC register) | No commonly misidentified lines were used. |

## Animals and other organisms

Policy information about studies involving animals; ARRIVE guidelines recommended for reporting animal research

| | |
|---|---|
| Laboratory animals | We used Drosophila melanogaster in this study. Only male adult flies were used for humidity-induced attraction behavior assays and antenna immnunostaining. Both genders were used for all other experiments. Genome edited strain generated in this study: Tmem63KO, Tmem63-LexA, Tmem63:EGFP. Transgenic strains generated in this study: UAS-DmTmem63, UAS-HsTmem63a, UAS-HsTmem63b, UAS-HsTmem63c. Other strains and sources: Bloomington Drosophila Stock Center: Or42b-Gal4 (9972), Or42b-Gal80 (80548), Nan-Gal4 (24903), Ir31a-Gal4 (41726), Ir41a-Gal4 (41749), Ir40a-Gal4 (41727), Ir75a-Gal4 (41748), Ir8a-Gal4 (41737), R11F02-Gal4 (49828), UAS-mCD8-RFP,LexAop2-mCD8:GFP (32229), LexAop2-GCaMP6m (44276), LexAop2-Gal80 (32213), UAS-Kir2.1 (6596), Orco-RFP (63045), UAS-RedStinger (8546, 8547), UAS-CD4-tdGFP (35836), UAS-GCaMP6m (42750), UAS-tdTomato (35837) and w1118 (3605). Vienna Drosophila Resource Center: UAS-Tmem63-RNAi (7363) and UAS-Dicer2 (60008, 60009). Ir68a-Gal4 from Knecht et al., Elife, 2017; nos-Cas9 and hs-Cre from Deng et al., Neuron, 2019. |
| Wild animals | The study did not involve wild animals. |
| Field-collected samples | The study did not involve samples collected from the field. |
| Ethics oversight | All animals were handled strictly in accordance with the guidelines of the Care and Use of Laboratory Animals of the National Institutes of Health and the Office of Laboratory Animal Welfare, China. The research protocol was approved by the Institutional animal care and use committee, Department of Laboratory Animal Science, Fudan University, China. |

Note that full information on the approval of the study protocol must also be provided in the manuscript.
